# Parallel Processing Strategies for Big Geospatial Data

**DOI:** 10.3389/fdata.2019.00044

**Published:** 2019-12-03

**Authors:** Martin Werner

**Affiliations:** Institute for Applied Computer Science, Forschungsinstitut CODE, Bundeswehr University Munich, Munich, Germany

**Keywords:** spatial computing models, cloud computing, spatial HPC, big data, MapReduce

## Abstract

This paper provides an abstract analysis of parallel processing strategies for spatial and spatio-temporal data. It isolates aspects such as data locality and computational locality as well as redundancy and locally sequential access as central elements of parallel algorithm design for spatial data. Furthermore, the paper gives some examples from simple and advanced GIS and spatial data analysis highlighting both that big data systems have been around long before the current hype of big data and that they follow some design principles which are inevitable for spatial data including distributed data structures and messaging, which are, however, incompatible with the popular MapReduce paradigm. Throughout this discussion, the need for a replacement or extension of the MapReduce paradigm for spatial data is derived. This paradigm should be able to deal with the imperfect data locality inherent to spatial data hindering full independence of non-trivial computational tasks. We conclude that more research is needed and that spatial big data systems should pick up more concepts like graphs, shortest paths, raster data, events, and streams at the same time instead of solving exactly the set of spatially separable problems such as line simplifications or range queries in manydifferent ways.

## Introduction

In the last decade, the term Big Data has been silently identified with web-scale cloud computing systems for handling big data. This is reasonable, because the big data movement was mainly initiated from Internet companies including Google, Facebook, and Twitter. For example, Google has made the MapReduce programming paradigm their default parallel system (Dean and Ghemawat, 2008, 2010) and has reached a wide audience with this. Facebook developed, for example, Apache Cassandra (Lakshman and Malik, 2010), and the HBase distributed database systems^1^ to solve their data management problem, most notably the inbox search problem (Lakshman and Malik, 2010).

While these problems are very beautiful and the software proposed and implemented is extraordinary powerful, the novelty and scalability of these systems is limited. The novelty of the MapReduce paradigm is not the structure behind: applying a function over data in a distributed system has been there long before. For example, Hudak mentions in his 1989 survey of functional programming (Hudak, 1989) that lists have been introduced as the core of functional programming by McCarthy to FLPL to be run on IBM 704 already in 1958 (Gelernter et al., 1960). In this survey, you will very often find the “mapcar” higher order function, which is essentially the first version of map. The name reduce is not very common in these early languages, as it stands for the central expression evaluation as well. However, folding is just the same as reducing and is a simple and common expression [see section 2.1 in Hudaks survey Hudak, 1989]:

   (fold f init) [ ] = init

   (fold f init)(x:xs) = f x ((fold f init) xs)

However, the popularity of MapReduce lies in the smart combination with block-based data locality and removing the burden of sorting data from the user in the shuffle phase.

These developments have been driven by the search for a scalable and cheap way of managing real-time data such as in social networks. On a more abstract level, the idea driving innovation was that faulty cheap computers can cooperate in order to create a scalable system for handling data at very low cost.

Interestingly, a significant number of researchers have started adopting this paradigm for research ignoring any alternatives (including, but not limited to functional languages like LISP, Haskell and R). Sometimes, these researchers even lock themselves into a single system and publish many papers adapting these architectures to the specific needs instead of architecting the ideal system for their needs. However, the needs of researchers are either completely theoretical (computer science, method development) or occasional (applied scientists). The first group usually works with very small clusters of sometimes even <10 nodes and the second class realizes that they will never have space and funding for the computing system of their need.

Most large universities and research bodies provide exceptional computing abilities with thousands of processing units to researchers for free in the framework of high performance computing (HPC) (Bergman et al., 2008; Lippert et al., 2011) and it has been shown that investment in HPC pays off for research competitiveness in general (Apon et al., 2010) and is likely to continue. As this technology has been around for decades, it is, however, not so eye-catchy as claiming having solved current problems with cloud-based software. While all major universities across the world can provide access to decent HPC systems, only very few of them provide significant cloud computing infrastructures. This means that researchers have to finance the hardware for their research on their own if they stick to cloud computing. And this leads to two aspects: first, papers about big data handle only a little bit of data and, second, compute clusters of <16 nodes are common in evaluating “big” data systems.

This paper discusses the challenges, opportunities, and pitfalls of big data systems from a more general perspective without going into individual systems or proposals. Instead, the author wants to collect the variations that distributed systems imply on the choice of indexing structures and on algorithm design. This position paper shall help to raise attention to the fact that all HPC systems are able to do big geospatial data as well and that—in my experience—at levels of performance that cannot be reached with cloud computing infrastructures at all and practically without costs to the research group. In addition, their nomadic and usually time-scheduled organizational structure makes them financially more efficient than distributed systems based on commodity hardware, because they contribute to results of a large group of researchers.

For the remainder of this paper, we will mostly focus on spatial and spatio-temporal data, which is significantly different from traditional big data workloads in that a sensible ordering of the data does not exist, which directly translates to a comparably higher amount of intra-cluster communication in distributed systems.

## Selected Aspects of Spatial Computing Design

Today, many different computing models are being used in the spatial domain, however, a discussion of their commonalities and differences is widely missing. For example, most of the traditional GIS and spatial computing research relies on some assumptions of the database community including that memory is organized into pages, algorithms are operating on these pages, indices should be compatible with the concepts of Generalized Search Trees (GiST) or Generalized Inverted Indices (GIN), consequently most of them being trees. Parallel execution and overheads implied by consistency demand of these data structures are widely ignored or pushed to the user level: a current database provides very fast access for many concurrent users and queries. Hence, it is parallel in a certain sense. However, keeping queries largely sequential objects operating on a snapshot of the data limits the scalability for individual queries significantly.

This tradition of database research brings many very interesting and very involved indexing techniques to life and helps in everyday work with spatial data a lot. Most often, the user itself is not working in parallel and the datasets that are being used are actually not that large at all. Hence, proposing GIS and even big data GIS people to start with a decent database management system like PostgreSQL with PostGIS is a valid position. However, these systems are usually tightly bound to the assumption that it is possible to maintain a single transactional scope for the whole data management process and, finally, this implies waiting times and degrades performance when scaling or with data that is quickly evolving or very huge. As the amounts of spatial observations are increasing in terms of resolution, frequency of observation, and accuracy, these traditional systems are limited if and only if the spatial problems are not easily separable into smaller independent pieces of data. If they are, we can just instantiate as many instances of a traditional database system as we need to solve our task. And this is actually heavily done in mapping and cartography, where high-resolution information is consumed only locally and never put into relation with highly-detailed data from far away.

In contrast to this rather traditional line of research, people have realized that some companies found themselves having to compute at a significantly larger scale in some of the following three dimensions: data volume, data velocity, and data variety. Large Internet companies including Google, Facebook, Twitter, and others, have then started to create their own highly distributed infrastructure in order to account for their business need which is serving millions of users with millions of changes everywhere in the world. From a systems perspective, these companies are in a very special situation which most research is not. They have millions of users essentially following some statistical access pattern leading to interaction parallelism. They have huge amounts of data and huge amounts of changes coming in. And they have the business need of permanent, fast and reliable service. In fact, the scale of these systems implied that it will be impossible to guarantee a good user experience with traditional techniques. The most specific limitation comes from maintaining consistency in evolving databases. It is known since about the year 2000, that a scalable system cannot be consistent, available, and partition-tolerant at the same time (Brewer, 2000; Gilbert and Lynch, 2002). What now basically happened is that these companies stepped back and implemented distributed systems holding such data dropping the ability to flexibly query data, the advantages of a relational design (e.g., limited redundancy, data consistency, and others). Nearly all of these big data systems are internally mapping to a key value store in which a single integer key is being used to distribute data across a cluster and to lookup data for requests. The main driver in this area is, however, financial scalability and tightly bound to concepts from cloud computing: The number of computers involved in the service can change at any time in any direction. Nodes may be added to increase performance, nodes may be removed to reduce costs or because they have failures.

These cloud computing systems are able to handle failures pretty well and, therefore, can exploit cheap hardware in a systematic manner. However, they are only efficient if the system utilization is sufficiently high. While this has led to nice pay-as-you-go models for compute, the limitation and problem is storage. If you want to store lots of data in the cloud, it gets expensive and you cannot share this resource. Finally, this means that if you don't need permanent compute, big data collections are very expensive to hold in a cloud computing data center. On the other hand, holding them locally, e.g., on tapes, is non-trivial and the initial population of the cloud computing system wastes computational resources.

As a third island and currently significantly underrepresented in the spatial domain, there is the area of HPC. In HPC, vendors build sophisticated systems for high bandwidth parallel computing optimizing for peak performance, usually without dynamic financial constraints. That is, given a certain space to set up a computer, a certain energy that can be made available, and a certain fixed amount of money, the design follows the rationale of building the fastest or most energy-efficient general-purpose supercomputer possible. These systems share many properties with cloud-computing based systems, for example, that they are highly distributed and that dynamic sub-clusters are usually assigned a certain task. However, there are some significant practical differences: These computers are usually time-scheduled and nomadic. That is, a researcher can submit a job to the system and wait for its execution, but he cannot run a long-running service or rely on any consistency properties of the cluster between different runs. Processing spatial data in such an environment is significantly different, because background maintenance work is usually possible only to a very limited extent.

Let us try to abstract from the specifics of these three worlds: sequential computing, cloud-computing, and high-performance computing. What are the common structures that could be used to guide algorithm and platform design?

The first and most obvious aspect is **data locality:** In all three cases, it is useful if data that is actually consumed together stays together (Zhang et al., 2015). For a traditional paging-oriented database, this means that accesses to the same database page should happen near enough to each other that the cache miss probability is kept low (DeWitt and Gray, 1992; Chung et al., 1995). In distributed cloud-computing systems, the slowest operation is to gather together some data that is stored on different computers relying on a usual data center network speed. Similar to paging in databases, one tries to avoid data transfer between different hosts and if it happens, we try to make most use out of any of these transmission before the temporary data is evicted from the machine that had to download it via the network. At first sight, HPC seems to be different: distributed file systems are in place which can be used to perform coordinated reads in excessive speeds and abstract away a lot of the hassle of data distribution. In addition, their I/O performance is so high that involved computations of spatial algorithms will actually take more time. However, these systems can exploit data locality one layer higher in the memory hierarchy: most of these systems are able to remotely read the main memory of a few nearby machines without interrupting the machine (e.g., remote direct memory access, R-DMA) (Liang et al., 2014). If our system is now able to keep related data near, then it could be that we can read it from remote main memory instead from disk giving significant performance gains.

In summary, data locality is a significant advantage in all three streams of computing research. Unfortunately, perfect data locality is impossible due to the scale and dimensionality of the data and, therefore, we need to implement and design data locality in a scalable way. In summary, we formulate the following design question: **if we have this dataset, this notion of locality, and this number of transactional scopes, each with a certain capacity, is it possible and how is it possible to distribute the data across the transactional scopes such that the locality notion is optimized?**

The second slightly less obvious aspect is redundancy: traditional relational database management systems avoid redundancy as much as possible simplifying write operations to the database and leading to clean data following a certain relational model and transactional isolation. Cloud computing, however, reaches numbers of computers in which the probability that a single computer will fail is too high to be ignored or managed (e.g., by carefully maintaining soft- and hardware in order to avoid any outages). Instead, outages are a normal behavior in such systems and the systems should self-heal themselves. The key to this is actually to increase redundancy to a level such that—starting from a healthy setup- a certain number of faults called redundancy factor can be tolerated and repaired (Wang et al., 1995; Shvachko et al., 2010).

A very simple strategy is to store all data on k different computers (or racks, data centers, …). Then, the outage of k-1 computers can always be tolerated, because it can make up to k-1 copies of the data inaccessible. The last copy of the data can be redistributed across the remaining cluster such that the system can actually heal itself toward the prescribed redundancy level (as long as enough computers are in a healthy state).

In current HPC systems, fault tolerance is not yet a default ingredient though the scale of these systems already suggests that it would be better to be able to handle faults. The upcoming next standard of the dominant Message Passing Interface (MPI) will include mechanisms to handle this situation (Fagg et al., 2004; Hursey et al., 2011). Until then, compute centers usually keep scheduled run times for HPC jobs sufficiently short and invest in hard- and software maintenance in order to avoid frequent node or processor failures. In addition, best practice for software development in HPC context is that interruptions should be expected and that software should be structured to maximize the effectivity of checkpoints that the system can store in order to continue a job that failed due to a local cluster outage.

In summary, the discussion of the previous section can be subsumed into the following design question: **given a dataset and a workload, what level of data and system redundancy is ideal, how do we create this redundancy, and can we write partial results (e.g., checkpoints) in order to alleviate otherwise critical faults**.

In this formulation, data redundancy is the question of how many independent copies of a certain data item are maintained, system redundancy is the question of how many nodes perform the same operation or are at least ready to take over the operation. It is worth noting that the ability to take over a certain operation implies the need to detect a malfunction and implies intra-cluster communication like heartbeats or other means of detecting a malfunction which are complicated in themselves.

In sequential systems, it is known that sequential access to data is usually faster than random access patterns. For spinning disks, this is related to the time needed to seek to a specific location, but even for modern SSD-based systems, the individual read operations usually read larger blocks of consecutive data. This is most often organized in the operating system and can be influenced through operating system APIs. Similarly, in database management systems, pages are read as an entire piece of information and can have a size of a few kilobytes to megabytes. In distributed file systems, files are as well split into larger pieces that can individually be managed (e.g., knowing which file they belong to). In summary, we can conclude that at least **locally sequential access** is a good I/O pattern for an algorithm implementation across all architectures. Many traditional disk-resident data structures imply a locally sequential access pattern. As they usually imply some sort of ordering of the data which is used to linearize the data on the disk, near data in the index will reside in neighboring pages or blocks. In a similar vein, algorithms can obey comparable access patterns. We call this aspect **computational locality**. While data locality and locally sequential access just means that near data is near in the index and physical data organization, computational locality means that the computation is organized in a way such that only near data needs to be related with each other. This means that a computation with data items A and B is only allowed if the distance of A and B is sufficiently small. If algorithms can be designed in this way, their execution can be significantly sped up. Furthermore, if problems can be formulated such that algorithms with this property exist, a lot of performance has been gained.

In summary, in this section we selected and formulated questions for designing big geospatial or spatio-temporal data processing systems, which can be used to classify many existing approaches and which can be helpful in designing and discussing algorithms in this field.

- **Data Distribution and Locality:** How to minimize data movement cost in distributed systems
- **Redundancy:** How to improve fault tolerance, resilience, and performance by data replication
- **Locally Sequential Access:** How do we exploit the fact that all read operations are reading blocks of data
- **Computational Locality:** How can we design problems and algorithms that they minimize the number of non-local data items needed?

These four aspects of the overall system are determining the reachable performance and provide room for tradeoffs and, therefore, for a certain variability in successful strategies.

## Morphology of Spatial Data

Traditionally, spatial data is only discussed in terms of its memory organization and representation in a single computer. In addition, a pure vector or pure raster representation model are used with few approaches actually being able to manage mixed data in high performance and quality (Maffini, 1987; Couclelis, 1992).

For the vector-oriented world, Simple Feature Geometry vector representations (points, polylines, polygons, polyeder, etc.), their topological predicates (within, overlaps, identical, intersects, etc.), and their core operators (Buffer, Transform, Extract, Envelope, …) are defined, implemented and used to express arbitrary cartographic and spatial data processes (Open Geospatial Consortium, 2007; Strobl, 2008).

In contrast, the raster-oriented world represents the spatial domain by first fitting and defining a grid to the world and processing features in terms of rank 3 tensors: Two dimensions of this tensor are being used for two main coordinates (latitude, longitude or X and Y) and the third rank is being used to model auxiliary data including a Z coordinate or other measurements. The study of these objects is usually done through local matrix operators like filtering and morphology, and through extraction of information on three levels: local, that is per pixel, focal, that is, in typically rectangular surroundings of pixels, and global, that is, across the whole raster (Tomlin, 1994).

This dominance of a purely algorithmic taxonomy of geometry has been challenged in the last decade mainly due to the rise of many more highly diverse data sources that do not really fit well into the pattern of either geometry or raster. Back in the past, the main data source for point data was actual geodetic measurements. People were walking around measuring points in relation to other points adding up to a database of measurements. Nowadays, however, many more data comes up naturally due advanced remote sensing, satellite positioning systems, and smartphones. While all of these create basically point-like information, the relation between different points is more explicit and usually needed for an application. For example, a spatial trajectory is represented by a certain set of points yet we assume that some sort of interpolation is essential to the concept of the trajectory. Or points generated from remote sensing systems are usually georeferenced with various sizes of errors. Still, inside the same sensor acquisition and image, errors tend to be comparable and smaller as opposed to an “absolute” error, which is not even easy to define given that the Earth is a highly dynamic object. Therefore, it is essential to know where the points came from and in what context they have to be understood.

In addition, the concept of places has been evolving (Schmid and Richter, 2006; Russell, 2008; Feld and Werner, 2013). Places are somehow bound to location, but not absolutely precise or unique in a geometric sense. In contrast, they are often defined as some clusters or aggregates of observed locations that are meaningful to the user. For example, “shop” might be a specification of a place and might include the area in front of the shop on sunny days as well. Or a public building like the city hall might be a place specification even without specifying the city. This becomes clearer in natural language sentences like “In the city hall, you can get your passport extended.” This sentence has a clear and specific spatial ingredient though it can only be resolved to a location together with the context of the conversation like which city actually is meant. However, it is universally true and not incomplete as a sentence.

An early and widely respected novel classification scheme has been proposed by Kuhn et al. under the term Spatial Core Concepts. They establish a scheme specifying “what spatial information is about, at a level above data models, but independent of particular application domain” (Dean and Ghemawat, 2010). Their scheme consists of six spatial concepts: Location, specifying a place in a geometric sense giving coordinates relative to a reference system giving sense to these numbers. Neighborhoods model the interaction of different locations mainly in the sense that neighborhoods contain things near to a location. Fields model phenomena that have a full spatial extent. That is attributes that exist everywhere and vary with time and space. Objects are bounded things in space having an identity possibly linking to a lot of data defining the object or its relations with other aspects. Networks cover connectivity and implied knowledge usually given using techniques from graph theory. Events, finally, are kind of a collective aspect summarizing changes to things representable with the other core concepts. This includes movement changing from one location to another as well as changes like objects appearing or disappearing.

In contrast to the traditional computational concepts of vector and raster graphics, the higher level of semantics of the involved concepts allows to define more specific algorithms and actually guide algorithm design. However, these core concepts are not sufficiently detailed if one wants to integrate current hot streams of spatial data research like trajectories. However, it is easy to follow the basic idea of these core concepts and to extend them to include additional concepts. Now, it becomes possible to clearly distinguish, for example, time series from trajectories though they share the same computational representation as a set of time-stamped points and as well many basic algorithms. And this can have a significant impact on polymorphism of functions.

Consider the simplification operation on both trajectories and time series, which shall be an operation that reduces the number of points. While the only way of simplifying time series is by leaving out some of the measured points (e.g., sampling a subset of the points of the time series), the interpolation assumption of trajectories allows different algorithms from the field of geometric simplification or even time-synchronous sampling in which a set of trajectories is sampled with locations at synchronous timestamps.

With this paper, we will not follow tightly to the spatial core concepts as proposed by Kuhn. However, we think that it makes sense to set up a taxonomy of spatial things that deserve significantly different algorithmic treatments even if they might share aspects of their representation in computing systems—just like trajectories and time series have identical representations, but different options for algorithms.

## A Classification of Computational Systems

In this section, we first classify computational systems in two dimensions, first, on the pure architectural implementation and, second, on the type of middleware that is used to coordinate especially parallel systems.

### Abstract Architectures

The previous two sections have set up two aspects for spatial big data algorithm design: first, we need to understand and learn on the aspects that dictate the practical performance of spatial algorithms in current and future highly distributed computing systems and, second, we need to take care that we learn to differentiate spatial concepts at a semantic level such that algorithms can be reused as much as possible across domains. This section recalls shortly four classes of real-world computing models and algorithm models that have significantly different properties.

A **Single Core Sequential Algorithms** is an algorithm that runs sequentially on a computer. Usually, it consists of a sequence of steps including loops, computations, and decisions turning an input to an output. Traditionally, sequential algorithms are discussed in terms of their worst-case performance, though this can be misleading as has been lately discussed in an ACM spectrum review article “Beyond Worst-Case Analysis” (Roughgarden, 2019). To this end, the database community has already established a widely respected set of benchmark datasets to augment such theoretical analyses with real-world performances. Toward big geospatial data, however, benchmark sets and workload types are currently widely missing, especially datasets crossing the historical boundaries of raster and vector graphics.

A **Shared Memory Parallel Algorithms with Atomic Operations** is an algorithm that runs on a multi-core system with a single main memory space. It runs in parallel and has the option of executing a certain subset of the operations in atomic form which means that the CPU cannot be interrupted by other concurrent activities. These features are common in modern CPUs and are needed to setup flags and to wait for conditions or to make sure that parallel execution does not destroy consistency of global variables. The advantage of shared memory programs is that they have a simple consistent joint state using global variables, but the downside is that their scalability is generally limited. However, machines with several terabytes of main memory are common in supercomputing centers, for example the TeraMem system of the Leibniz Rechenzentrum in Munich^2^.

A **Distributed Memory Parallel Processing Algorithm** is an algorithm designed for a system in which independent distributed components perform a joint task without any consistently shared resource. The computers or nodes are connected via a network and coordinate their work by communication. The assumptions on the communication are usually pretty vague like it is guaranteed that each message is received at least once, but with no guarantees on when this happens or even on whether it happens exactly once. In general, the coordination of such systems is tricky and comes in two major flavors: introducing a (logically) central management component as is routinely done in cloud computing systems (e.g., the NodeManager in Hadoop) and HPC systems (e.g., MPI rank zero) or introducing a set of rules that all distributed components obey in order to reach a consistent joint result.

As it is difficult to apply complexity theory to arbitrary distributed algorithms, it is common that algorithms designed for both shared memory multiprocessing and distributed memory parallel processing are evaluated only on synthetic and real-world workloads using wall-clock time or energy consumption as the main metrics of interest and that an evaluation in terms of theoretic bounds is not performed. An interesting example of how this is done, is given by a paper describing how to scale deep learning to the (currently) fastest supercomputer in the world including 27,600 GPUs (Laanait et al., 2019).

### Middleware and Framework

Many programs written in these various computational environments share an outer structure and we want to list the most important of these shared structures as one expects that highly optimized such basic structures form a sensible building block for real-world algorithm design and implementation.

The Message Passing Interface (MPI) is a traditional middleware solution for distributed memory parallel processing and most (if not all) high-performance computing (HPC) systems support optimized versions of MPI as the distributed computing framework. In MPI, a set of computers is collected into a communicator and all nodes are numbered from zero to n-1 called rank. Now, communication and coordination relies on message passing, that is, it is possible to send messages between those nodes. Communication in MPI is in general either synchronous or asynchronous and can be collective or point-to-point. But the basic idea of MPI is to provide highly optimized implementation of common problems in HPC environments like reading data from a distributed file system, distributing data across the cluster, reducing and aggregating results, and managing input and output of the system. Therefore, the MPI system provides high-level API functions for data distribution and synchronization (Barker, 2015; Hoefler et al., 2015; Garrett, 2017).

Another similarly traditional approach from the field of distributed systems is the Remote Procedure Call (RPC). An RPC is a mechanism with which it is possible to invoke a function on a remote system as if it was a local procedure. In fact, most libraries for RPC represent it actually as this: a middleware generates an implementation of a local function that first sends the parameters over the network to a remote host, invokes a subroutine there, and collects the results and returns them. That is, for the program using an RPC, it feels like a fully local function call. Traditional RPCs have been around for long, for example in the context of CORBA (Hudak, 1989), but the framework of RPC is an ingredient to many current systems including gRPC (Gelernter et al., 1960). Current web services are largely request-based and a request can be seen as a remote method invocation. However, they extend the framework of RPC with aspects such as attaching to streams and reacting to events.

In terms of big data, novel middleware approaches have been evolving which limit the interface and structure of functions that can be used. While the previously mentioned approaches are independent of the structure of the program, a novel paradigm of structuring distributed computing has been proposed with the MapReduce framework. In a MapReduce system, the dataset is distributed in the beginning over the set of involved nodes (Dean and Ghemawat, 2008; Hashem et al., 2016). Then, a map function can be applied over the data which means that it is invoked once for every data item (like a line in a text file or a record in a dataset). This map function is able to consume data and it is also able to produce and emit data to the next stage. In general, data is represented in a key value fashion such that every data item has a key associated with it. This becomes important in the next phase: after a map operation has been invoked for all data items and has generated named data items as the output, these data items are sorted by the middleware and re-distributed across the cluster such that the Reduce function is invoked once for each key that has originated somewhere in the distributed system with all data items that have been generated with this key.

In addition, the Reduce function can have properties like associativity and commutativity such that the middleware can interleave the execution and communication of the Reduce function (this is sometimes called a Combiner if it is run on each distributed node before the data is shuffled across the network bringing together all data with the same key). This fixed structure has been popularized by Google (Dean and Ghemawat, 2008) and has been implemented as open source in the Hadoop project (Lippert et al., 2011). It has after that been extended to allow for more flexible patterns and to include in-memory processing capabilities in the project Apache Spark (Bergman et al., 2008).

## Implementation Patterns and Strategies

The complicated and highly diverse computational environment today as described in the previous sections often hinders the design of optimal systems. In many practical cases, there are real-world constraints coming from history (things you already know, own, or have access to) or from the behavior of the crowd (cloud computing is a hot topic). In this section, we want to collect the most abstract algorithmic patterns that can be implemented in all combinations of the previously mentioned aspects including hardware, frameworks, and spatial data conceptualizations.

In general, there are two strategies to turn a computing system from sequential to parallel: Data-parallel and Task-parallel. Loosely speaking, a data-parallel system does the same thing to different data while a task parallel system creates a list of independent tasks which are executed in parallel.

Data parallel systems are easily supported in hardware, for example with SIMD instructions. These are instructions that do not work on a single register or pair of registers, but rather on a small “array” register. For example, in a current 64-bit architecture, SIMD units have 128 bit, 256 bit or more bits and can do a subset of the operations (e.g., adding, multiplying, etc.) on these large registers in a single CPU instruction. This finds wide application in numeric calculations (e.g., matrix multiplication), but even in text parsing. For example the RapidJSON and simdjson JSON parser rely on SIMD instructions to skip over multi-byte patterns in a single step reaching gigabyte/s parsing speed for JSON documents (Langdale and Lemire, 2019).

Of course, such hardware extensions support only a very limited number of algorithms completely, therefore, data-parallel systems can be implemented using threads or processes as well. In a data-parallel thread system, a pool of threads applies the same “thing” over the data. A typical example is a parallel for loop in which the body of the four loop relates to a section of the input data and produces a section of the output data. If we can guarantee that the sections of the output data are disjoint, we can let threads run without any synchronization. If the outputs can overlap, we have to make sure that the order in which the bodies of the loop generate results does not matter to the results. This often implies synchronization making sure that only a single thread is able to write a single block of memory at any time.

Task-parallel systems are often implemented as a task queue. In this setting, a set of tasks is ordered into a queue and—depending on certain preferences or heuristics—the involved compute nodes will pull a task from this queue, execute it, and publish or store the result. A more involved task-parallel system is a divide-and-conquer system in which the distributed task queue starts with a few tasks only and the tasks are split into smaller subtasks which are then further distributed across the cluster.

As already outlined, the simplest solution of parallel programming is to have many tasks perform independent operations, because this avoids the need of synchronization and—depending on the flexibility or scale of the number of tasks—makes it easy to execute tasks in parallel.

A little bit more involved is the case when many tasks are **loosely coupled to a joint objective**, for example, the application purpose. In this case, the tasks are “technically” independent and can be run in parallel without any synchronization, but there is a higher layer of synchronization and consistency management which reduces the parallel efficiency in some situations. A typical example is the MapReduce paradigm: While the Map-phase consists of independent tasks that take a subset of the input data and contribute to the output of the Map phase without any communication between tasks, the precondition of the Reduce phase is that the Map phase has finished or it is otherwise guaranteed that all elements that end up in the same Reducer are already available. In practice, this very aspect is organized by a central entity called Master, but it is not a significant scalability bottleneck, because the master does not actually manage the data, but rather the task metadata: which tasks have been submitted to which node and which tasks have already completed.

Even more involved is the case of pipelines, though they actually create the highest possible parallel speed. In a pipeline system, a sequence of tasks must be applied to the data and each of those tasks is executed by another thread and the demand is communicated: the successful execution of a task triggers the next task in the pipeline on completion. This pattern is known as stream computation and Apache Flink or Apache Storm are respected implementations of this pattern (Hesse and Lorenz, 2015). The advantage of this is that waiting can be avoided in many cases leading to a higher efficiency. In addition, it is easy to scale by adjusting the number of threads that take over certain tasks.

The **producer-consumer** pattern is a mixture of client-server and pipeline patterns. Each component can act as a producer and as a consumer and all producers and consumers form a graph in which information (e.g., tasks and/or data) flow from producers to consumers. A special producer-consumer pattern is the pattern of distributed task queues. In these, each node has a local task queue which acts as a producer and a thread pool which consumes tasks from this queue. However, each task can produce new tasks locally and remotely such that the consumer turns to a producer in certain aspects. Finally, the program ends when no producer generates new tasks or new data.

## Application to Big Geospatial Data

The previous sections have collected the needed background to present a framework for designing scalable algorithms for big geospatial data. In this section, we will discuss a certain set of spatial algorithm classes and how they fit into the diverse categories of big data computing systems and frameworks.

The basic function of any GIS or spatial database is to enable the user to access spatial subsets of the data for further processing. Three types of queries are typical in this area:

- **Range Queries:** To find the objects in a specified spatial range (e.g., circle, MBR, polygon).
- **Nearest Neighbor Queries:** To create an ordered list of k items in increasing distance.
- **Spatial Join Queries:** To compute the spatial predicate interactions of two datasets.

For all of these queries, spatial indices are routinely used in traditional computing. As we already explained, data locality is key to scalability and we need to set up data locality patterns such that physically nearby things (those that fulfill the same query predicates with high probability) are near each other.

If the data is not changing significantly or if the spatial data distribution is known, the best approach will be to grow some recursive spatial indexing tree like an R-tree using sort-tile-recurse (STR) bulk loading until a certain number of nodes has been created. For each of those nodes, a task is created which is to solve the range query for all data that belongs to this node. If the spatial indexing tree is sufficiently balanced or if the tree is grown until the task size is comparable, a task parallel system has been defined in which data locality comes from a spatial indexing tree. If the queries that are processed in the system are similarly distributed as the data, this system will generate a high parallel efficiency (Eldawy and Mokbel, 2015). However, if the queries are sparse and local, the systems main limitation lies in the fact that due to data distribution only a few nodes can contribute to answering a single query, namely those that have the relevant data locally. If the workloads are, however, skewed against the spatial distribution of the dataset, two strategies can be followed: to implement redundancy increasing the number of nodes that own specific data until the capacity of the distributed system is exceeded. This can be done in a random fashion or following a different indexing and ordering scheme, for example, from time-intervals. The goal is to minimize the amount of compute nodes that are needed to answer a query while maximizing the amount of nodes that could sensibly contribute to answering a query.

While many systems follow the data distribution (e.g., Kini and Emanuele, 2014; Whitman et al., 2014; Eldawy and Mokbel, 2015; Xie et al., 2016), it has not yet been widely discussed how to follow the query distribution or how to adapt to the query workload during execution. This is an interesting direction for spatial big data research: How can we actually exploit the joint distribution of queries and data in distributing data across the cluster to solve the tradeoff between query locality and the number of nodes that could contribute to a query execution.

A second category of queries is the category of **Basic Topology Queries**. These include, for example,

- **Shortest Path Problems:** Find shortest paths between vertices of a graph.
- **Traveling Salesman Problems:** Given a distance matrix between k objects, find the tour across all of them that minimizes the total distance traveled.
- **Connected Components:** Compute connected components of the underlying graph.

These problems are typically solved by applying graph search algorithms and their variants over a graph. A widely-used data structure for efficient representation graphs is an adjacency list. In this context, the vertices are modeled and together with each vertex, a list of the outgoing edges (and sometimes as well a list of the incoming edges) is stored. A typical approach to parallel graph algorithms is to distribute this adjacency list across a cluster and to run algorithms across the global graph. This might imply that algorithms run across a different set of computers in order to solve a certain problem, especially, when following the out-edges crossing node boundaries. An MPI implementation has been proposed with the Parallel Boost Graph Library PBGL^3^. It is interesting to look in detail into this implementation as it provides certain program and data structures that come in handy when designing distributed data structures in an MPI setting. For example, they implement triggers, which can be used to asynchronously send messages to remote data structures. In a certain sense, these triggers can be used to invoke a member function of a distributed data structure on a remote node, a variant of RPC inside the framework of MPI. In addition, a distributed queue has been implemented which is a view of a set of local queues. Each node executes the elements from a local queue. But this execution can push data to a remote queue allowing for the implementation of various parallel algorithms and the exploitation of remote direct memory access.

From an indexing point of view, it is, of course, possible to use a spatial index for a spatial graph in order to distribute the adjacency list across the cluster improving locality. If the graph is not embedded into a Euclidean space, such a geometry can be derived from the topology of the graph through embeddings such as T-SNE (van der Maaten and Hinton, 2008). In Euclidean graphs (or in graphs with a synthetic Euclidean geometry attached), landmarks can be interesting in which a Dijkstra search is run from a certain set of nodes for a predefined depth or distance. Landmarks are added until the whole graph has sufficing landmark coverage. Then, search algorithm can quickly prune directions using a variant of the triangle inequality. One example of this class is ALT search (Goldberg and Harrelson, 2005) which has won the ACM SIGSPATIAL GIS Cup 2015 in a shared memory multiprocessing setting for dynamic street networks (Werner, 2015). However, parallel topology computing has not been widely discussed in the spatial computing domain and offers various options for future research.

The traveling salesman (TSP) type of graph problems stands out because these problems are known to be NP-hard. However, an approximation scheme has been defined for Euclidean TSPs allowing for efficient and effective calculation of the exact solution of the traveling salesman problem exploiting the triangle inequality. But, in general, good solution for the TSP can also be generated using heuristics such as local search or genetic optimizations (Korte et al., 2012). While these are naturally parallelizable, it is difficult to exactly know the quality of a solution. Parallel computing and TSP problems is, however, a very active research area (Zambito, 2006). The difficulty of solving even medium-sized problems [e.g., the world TSP^4^ is only 11.5 megabytes or 1,904,711 points, but an exact solution has not been proven] motivates a lot of research and might be one of the domains in which quantum computing might provide a revolution (Srinivasan et al., 2018).

However, more research is needed to solve spatial versions of real-world instances of the Traveling Salesman Problem in acceptable time using distributed computing. Instances of interest will be much smaller than the two-million city example and they might have additional structures like partial orderings that could be exploited to solve the problem or to generate approximate solutions quickly.

The third category for spatial computing operations is a category of geometry operations actually changing or generating geometry. Representative examples of this category of operations are

- **Simplification:** Given a geometric object, represent a sufficiently similar object with fewer data points.
- **Buffer:** Given a geometric object, create an enlarged version of this object.
- **Skeleton:** Given a geometric object, create a smaller version of the object.

These algorithms can be parallelized quite easily, because all of them are local. For example, if we need to simplify a huge geometric object, we can split the object into smaller pieces and simplify those pieces. For raw simplification, no synchronization is needed, in some cartographic scenarios, however, we need to track that the simplification process does not change the topology of the object. For example, a line simplification of a river must not lead to the situation that a city is depicted on the wrong side of the river after simplification. It is worth noting that simplification is a complex topic and usually involves algorithms of non-linear runtime. The most traditional algorithms, Douglas Peucker, works on linestrings or rings in a divide and conquer approach as follows: The first simplification is the line connecting start and end point. Then, the point with a largest error measure is found, inserted into the result, and used to split the problem into two sub-problems before and after this inserted point. Douglas Peucker algorithm is then recursively applied to all such divisions forming a tree of computations until the simplification fulfills the given error bound everywhere. The worst-case running time of this approach is quadratic in the number of points and the best algorithm known has a worst-case complexity of O(n log(n)) and is based on geometric hulls of the paths (Hershberger and Snoeyink, 1992). This is a beautiful and traditional spatial big data example as it exploits the spatial structure of the problem in order to make larger instances feasible. Given that this paper was already published in 1992, this highlights that spatial big data is significantly older than the big data movement of the last decade. Similarly, the buffer operation which enlarges a geometric object is naturally parallelizable, but needs careful design of synchronization, because the buffer shall be a consistent object (e.g., a polygon) and the generated part shapes might be significantly influenced by geometry that has not been available in a natural subdivision of the data. One algorithm was proposed optimizing load-balancing by Dong et al. (2003) and is an interesting read to get into this domain of parallel geometry processing algorithms.

Many more algorithm categories can be defined, but this paper is not intended to become a review of parallel geometry processing. Instead, we want to use the already-presented aspects to come back to the main topic of what structures algorithm designers should look for in order to find efficient variants for spatial processing operations. Abstracting from the walk-through of a representative set of GIS problems and options for their parallel implementation, we now try to isolate some abstract aspects of the presented approaches which might guide algorithm development.

From the first category of basic GIS functions, we conclude that the statistical distributions of the input data and the distribution of this input data across computational units is a very difficult question and needs to be investigated in detail, because there cannot be a good solution for many workloads. If the data distribution across the cluster ensures very good data locality, most queries will suffer from computational locality, that is, only a small fraction of the cluster has access to the data needed to answer the query. If on the other hand, the query distribution is taken as the design rationale, the data distribution might be heavily skewed leading to subtasks of different complexity across the cluster in cases where the data and query distribution do not coincide. In many cases, however, some structures of the data locality pattern are shared across queries and data, especially when it comes to data that is correlated to the same third distribution like population density. Therefore, data scientists working with huge sets of spatial data should look at the joint distribution of queries and data.

From the second category of GIS functions, those related to graph theory and topology, we conclude that there are many algorithms in which the computation will not be local after splitting the data across nodes. For the graph search, this means that a shortest path search will walk around the cluster and that we need a lightweight mechanism of invoking remote methods on a distributed data structure. A distributed queue in the semantics of the parallel boost graph library is a very clean and powerful tool, because it allows to have a clear notion of computational responsibility (e.g., the local queue) while allowing for distributing work across the cluster without a central entity (e.g., by triggering functions on remote nodes). This is significantly different from the implementation structure of many open source big data stacks, which usually follow a master-slave paradigm with a central component limiting their scalability. However, finding out whether such an algorithm terminated can become difficult, because we have informally written that the algorithm terminates if no thread produces new data. How do we know? This is a matter of debate and needs a master node again, this time only to collect one bit per node, namely, that it is not going to generate new tasks. However, in large systems, this one bit can be reduced by a collective Reduce operation such that it is compressed on its way to the master node.

From the third category of geometry operations, we remember that geometry often allows for a natural divide-and-conquer structure (e.g., splitting a raster into cells, splitting a polygon into triangles, splitting a linestring into sub-linestrings) with varying amounts of synchronization needs. For Douglas Peucker, synchronization is easy as all subtasks are independent, for the geometric buffer operation, however, the results of the subtask must fit to each other and the amount of geometric context needed to calculate the buffer in a location is not known. Complex distributed data structures with some synchronization mechanisms are the consequence and paradigms such as MapReduce are non-trivial to apply to these problems.

## Conclusion

With this paper, we first gave an overview of the computational infrastructures that are available today. We set up some intuitive questions that can guide algorithm design including data distribution and locality, redundancy in distributed systems, locally sequential access (also known as cache-awareness) and computational locality (that is, that algorithms rely on local data). While these intuitive measures are helpful, they are not precise enough to guide algorithm design. Therefore, we discuss both available middleware for computing as well as common structures for parallel programs. With this background information, we discuss as examples three classes of basic spatial and condense the central design patterns out of these. These are, first of all, data distribution, query distribution, data locality and computational locality. The second aspect is the question, what happens if data locality is possible, but computational locality is not. A basic example is shortest path search in large graphs. While we can split the graph across nodes, we cannot make sure that all paths reside on a single node. Instead, the graph search will move across the graph and, thus across the cluster. For scalable graph algorithms, this has been implemented in a messaging paradigm of unlimited scalability by implementing distributed queues and functions to synchronize the emptiness of this distributed queue and of submitting new information to these queues. Finally, we show that spatial data has a natural divide and conquer structure (e.g., by space subdivisions), but that a coordinated computation is not always easy, that is, a distributed algorithm for spatial data must include methodology to exploit non-local context and to ensure final global consistency of the results.

In summary, this paper showed that even a very basic GIS, as soon as it leaves the area of pure range and nearest neighbor search, is not directly compatible with MapReduce and that much more advanced structures from distributed computing including triggers and distributed queues of varying types are needed to implement distributed algorithms. An interesting and ultimately useful research direction would be the question whether there is a generalization of the strict independence assumption of MapReduce allowing for a wider class of spatial problems to be computed in the framework. In addition, we wanted to highlight, that traditional HPC and big data processing is a valid and interesting direction and that the community should start to investigate the actual usefulness of cloud computing given that HPC infrastructures are widely available to science for free (based on a scheme of applications guided by scientific excellence) while large-scale cloud computing is not yet widely available and expensive. Finally, many algorithms from spatial computing do not have rock-solid and system-agnostic distributed implementations making it impossible to reliably compare different approaches from an algorithmic or practical point of view. Therefore, both the development of benchmark dataset collections with a good workload coverage as well as the design of a more abstract spatial computing framework seem to be needed to combat the current fragmentation of contributions given the fragmented computational environment.

## Author Contributions

MW is the only contributor to this manuscript.

### Conflict of Interest

The author declares that the research was conducted in the absence of any commercial or financial relationships that could be construed as a potential conflict of interest.

## Footnotes

^1^Apache HBase – Apache HBase™ Home. [Online]. Verfügbar unter: https://hbase.apache.org/. [Zugegriffen: 23-Sep-2019].

^2^LRZ. *Teramem System for Applications with Extreme Memory Requirements*. Available online at: https://www.lrz.de/services/compute/special_systems/teramem/

^3^Edmonds, N., Gregor, D., and Lumsdaine, A. *The Parallel Boost Graph Library*. Available online at: https://www.boost.org/doc/libs/1_63_0/libs/graph_parallel/doc/html/index.html

^4^University Waterloo. *The World TSP*. Available online at: http://www.math.uwaterloo.ca/tsp/world/
